# A Case of Syncope

**DOI:** 10.5811/cpcem.2017.4.33725

**Published:** 2017-10-03

**Authors:** Kristin H. Dwyer, Joshua S. Rempell

**Affiliations:** * Brown University, Warren Alpert School of Medicine, Department of Emergency Medicine, Providence, Rhode Island; † Cooper Medical School of Rowan University, Cooper University Hospital, Department of Emergency Medicine, Camden, New Jersey

## CASE PRESENTATION

**Image 1:**
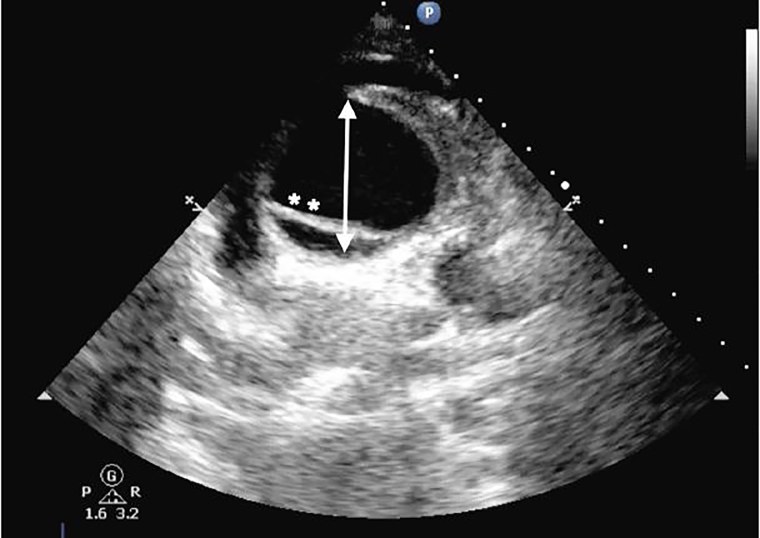
Suprasternal notch short-axis view performed on point-of-care ultrasound in elderly patient presenting to the emergency department after a syncopal event; aortic arch (arrow) with dissection flap (stars).

An 88-year-old female presented to the emergency department (ED) after a syncopal event. Upon arrival, the patient was awake and complaining of chest pain. An electrocardiogram was performed showing an inferior ST-elevation myocardial infarction (STEMI). Patient’s vital signs were heart rate of 86 beats/minute, blood pressure of 83/50 mmHg, temperature of 98.8 degrees Fahrenheit, respiratory rate of 18/minute, and oxygen saturation 96% while breathing room air. Dorsalis pedis pulses were difficult to appreciate bilaterally and the patient was agitated and diaphoretic. A focused cardiac ultrasound (FOCUS), including a suprasternal notch view (SSNV), was performed (Image 1).

## DIAGNOSIS

Ascending aortic dissection (AAD) is a lethal disease that is often misdiagnosed and commonly referred to as the “great masquerader.” Symptoms are often vague, and mortality increases 1–2% per hour with delays in diagnosis.^1^–^2^ Studies have shown that ED providers are able to identify AAD on FOCUS.^3^–^4^ Rarely, an AAD presents as a STEMI, and if treated with thrombolysis most patients will die from hemorrhagic complications.^5^

**Image 2:**
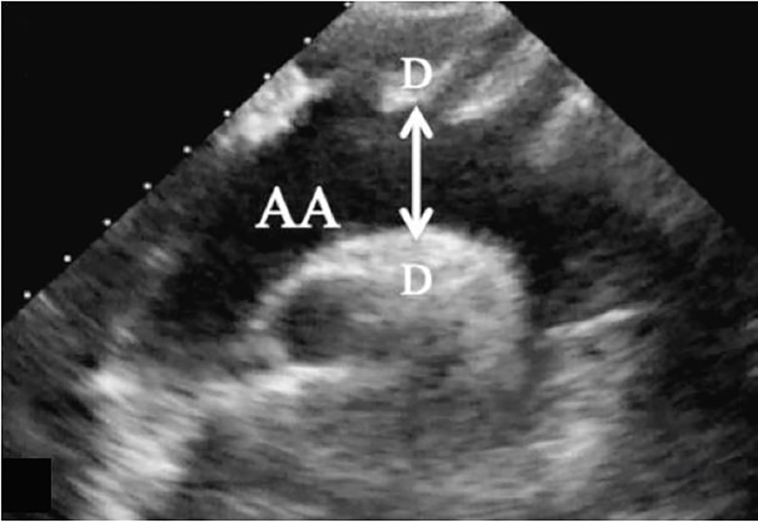
Normal suprasternal notch long-axis view. *AA,* aortic arch; *D*, widest diameter of aortic arch.

**Image 3:**
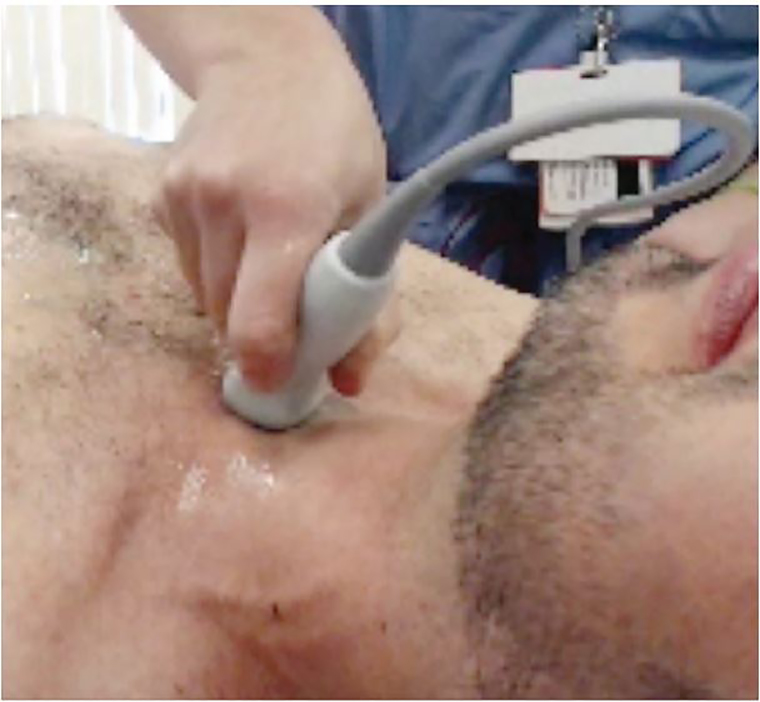
Probe positioning for suprasternal notch view with probe indicator pointing towards the patient’s right hip.

While not commonly performed in the ED, SSNV permits visualization of the aortic arch and the origins of the innominate, left common carotid, and the left subclavian arteries (Image 2).^3^ It has been shown to be easily obtained by emergency physicians with basic training. Diagnosis of a dissection is suggested by visualization of a flap in the aorta on ultrasound. Ascending aortic dissection is also associated with aortic dilation greater than 4cm.^6^ The technique involves placing a phased array transducer in the suprasternal notch with the indicator aimed toward the patient’s right hip (Image 3).

Use of bedside ultrasound SSNV upon patient arrival resulted in early diagnosis of AAD prior to initiation of anticoagulation and travel to the catheterization lab. The thoracic surgery team was activated to come into the hospital based on this image.

**CPC-EM Capsule**

What do we already know about this clinical entity?

*Ascending aortic dissection is a vascular emergency with significant associated mortality. The diagnosis is time sensitive as well as difficult to make. While pathology of the abdominal aorta is commonly evaluated for by the emergency provider, fewer are using point-of-care ultrasound (POCUS) to evaluate aortic pathology in the chest. Sometimes an inferior* ST-elevation myocardial infarction (*STEMI) can be the result of an ascending dissection, which involves the right coronary artery, and the treatment for these two disease entities is very different. The aortic arch can be visualized using the suprasternal notch view and can be identified by emergency providers with basic training.*

What is the major impact of the image(s)?

While not every STEMI patient needs an echocardiogram at bedside prior to cardiac catheterization, a dissection may be suspected in some patients with an inferior STEMI. Suspicious features may include pain radiating to the back, syncope, decreased pulses or hypotension. Computed tomography prior to catheterization for inferior STEMI is not the usual or appropriate course of action; however, heparinizing a patient with an ascending dissection and sending him to the catheterization suite will increase mortality.

How might this improve emergency medicine practice?

It is important to remember that a small percentage of patients presenting with an inferior STEMI may be having an ascending dissection. In those patients for whom you have a high degree of clinical suspicion, consider a POCUS to evaluate the aortic arch. This view can be obtained at the bedside in seconds and dramatically change the course of treatment.
